# Endoscopic endonasal resection of craniopharyngiomas: a case series and review of the literature

**Published:** 2012-11

**Authors:** Mohammad Samadian, Nader Akbari Dilmaghani, Seyyed Hadi Samimi, Habibollah Moghaddasi, Navid Farzin, Shahab Khazanedari

**Affiliations:** ^*a*^Neurosurgery And ENT wards of Loghman Hakim Hospital, Shahid Beheshti University of Medical Sciences , Day General Hospital, Tehran, Iran.

## Abstract

**Background:**

Transcranial approaches were historically the first established routes for craniopharyngiomas resection. Transcranial approaches commonly entail some degree of brain retraction as well as manipulation of neurovascular structures, located between the surgeon and the pathogenic region. A more direct route to the sellar -suprasellar region is provided by the transsphenoidal approach. Introduction of extended approaches offered a safe alternative technique for reaching suprasellar craniopharyngiomas. The next stage of development was incorporation of the endoscope in the extended approach which increased the field of view and illumination. There is a limited body of literature on the outcome of endoscopic endonasal approach in resection of craniopharyngiomas. Therefore, the present study aims to assess the outcome of endoscopic endonasal approach in resection of craniopharyngiomas.

**Methods:**

Eight patients underwent surgery at Loghman-Hakim and Day General Hospitals (Tehran, Iran). One patient had recurrent lesion. The gross-total resection (GTR) was attempted in 6 surgeries. Indications for intended subtotal resection included advanced age, medical comorbidities, preservation of pituitary function, and hypothalamic invasion.

**Results:**

The average size of tumor diameter was 2.8 cm. GTR in 6 cases and near-total resection in 2 cases were achieved. The average follow-up period was 30 months during which one recurrence in near resection cases was observed. Vision was improved by 80%. Four cases developed diabetes insipidus (unrelated to diabetes mellitus) and 3 cases developed panhypopituitarism postoperatively. Furthermore, postoperative cerebrospinal fluid (CSF) leaks occurred in 2 patients, one of them needed reoperation for CSF leak management. In addition, postoperative bacterial meningitis occurred in one patient.

**Conclusions:**

Endoscopic, endonasal surgery for craniopharyngioma can be accompanied by high rates of GTR with low rates of CSF leak.

**Keywords:**

Craniopharyngioma, Endonasal, Endoscopic


					Volume 4, Suppl. 1 Nov 2012
Publisher: Kermanshah University of Medical Sciences
URL: http://www.jivresearch.org
Copyright:
Congress of Iranian Neurosurgeons, 14-16 November 2012, Kermanshah, Iran
Paper No. 22
Endoscopic endonasal resection of craniopharyngiomas: a
case series and review of the literature
Mohammad Samadian a,*
, Nader Akbari Dilmaghani a
, Seyyed Hadi Samimi a
, Habibollah Moghaddasi a
,
Navid Farzin a
, Shahab Khazanedari a
a
Neurosurgery And ENT wards of Loghman Hakim hospital, Shahid Beheshti University of Medical Sciences , Day General
Hospital, Tehran, Iran.
Abstract:
Background: Transcranial approaches were historically the first established routes for craniopharyngiomas resection.
Transcranial approaches commonly entail some degree of brain retraction as well as manipulation of neurovascular
structures, located between the surgeon and the pathogenic region. A more direct route to the sellar -suprasellar
region is provided by the transsphenoidal approach. Introduction of extended approaches offered a safe
alternative technique for reaching suprasellar craniopharyngiomas. The next stage of development was incorporation
of the endoscope in the extended approach which increased the field of view and illumination. There is a limited
body of literature on the outcome of endoscopic endonasal approach in resection of craniopharyngiomas. Therefore,
the present study aims to assess the outcome of endoscopic endonasal approach in resection of craniopharyngiomas.
Methods: Eight patients underwent surgery at Loghman-Hakim and Day General Hospitals (Tehran, Iran). One
patient had recurrent lesion. The gross-total resection (GTR) was attempted in 6 surgeries. Indications for intended
subtotal resection included advanced age, medical comorbidities, preservation of pituitary function, and
hypothalamic invasion.
Results: The average size of tumor diameter was 2.8 cm. GTR in 6 cases and near-total resection in 2 cases were
achieved. The average follow-up period was 30 months during which one recurrence in near resection cases was
observed. Vision was improved by 80%. Four cases developed diabetes insipidus (unrelated to diabetes mellitus)
and 3 cases developed panhypopituitarism postoperatively. Furthermore, postoperative cerebrospinal fluid (CSF)
leaks occurred in 2 patients, one of them needed reoperation for CSF leak management. In addition, postoperative
bacterial meningitis occurred in one patient.
Conclusions: Endoscopic, endonasal surgery for craniopharyngioma can be accompanied by high rates of GTR with
low rates of CSF leak.
Keywords:
Craniopharyngioma, Endonasal, Endoscopic
* Corresponding Author at:
Mohammad Samadian: Neurosurgery And ENT wards of Loghman Hakim hospital, Shahid Beheshti University of Medical Sciences, Day General
Hospital, Tehran, Iran. Tel: 09123262849, Email: mdsamadian@hotmail.com, (Samadian M.).

				

